# Simplified Risk Stratification Model for Patients With Waldenström Macroglobulinemia

**DOI:** 10.1200/JCO.23.02066

**Published:** 2024-05-24

**Authors:** Saurabh Zanwar, Jennifer Le-Rademacher, Eric Durot, Shirley D’Sa, Jithma P. Abeykoon, Patrizia Mondello, Shaji Kumar, Shayna Sarosiek, Jonas Paludo, Saurabh Chhabra, Joselle M. Cook, Ricardo Parrondo, Angela Dispenzieri, Wilson I. Gonsalves, Eli Muchtar, Sikandar Ailawadhi, Robert A. Kyle, S. Vincent Rajkumar, Alain Delmer, Rafael Fonseca, Morie A. Gertz, Steven P. Treon, Stephen M. Ansell, Jorge J. Castillo, Prashant Kapoor

**Affiliations:** ^1^ Division of Hematology, Department of Medicine, Mayo Clinic, Rochester, MN; ^2^ Division of Clinical Trials and Biostatistics, Department of Quantitative Health Sciences, Mayo Clinic, Rochester, MN; ^3^ Department of Hematology, University Hospital of Reims and UFR Médecine, Reims, France; ^4^ University College of London, London, United Kingdom; ^5^ Dana-Farber Cancer Institute, Boston, MA; ^6^ Division of Hematology, Mayo Clinic, Scottsdale, AZ; ^7^ Division of Hematology, Mayo Clinic, Jacksonville, FL

## Abstract

**PURPOSE:**

Patients with Waldenström macroglobulinemia (WM) have disparate outcomes. Newer therapies have emerged since the development of International Prognostic Scoring System, and *MYD88*^*L265P*^ mutation is now frequently assessed at diagnosis, warranting reexamination of the prognostic parameters.

**PATIENTS AND METHODS:**

We reviewed records of 889 treatment-naïve patients with active WM, consecutively seen between January 01, 1996, and December 31, 2017, to identify clinical predictors of overall survival (OS) in univariate analyses. Patients with complete data for the parameters significant on the univariate analyses (n = 341) were included in a multivariable analysis to derive a prognostic model, subsequently validated in a multi-institutional cohort.

**RESULTS:**

In the derivation cohort (n = 341), age (hazard ratio [HR], 1.9 [95% CI, 1.2 to 2.1]; *P* = .0009), serum lactate dehydrogenase (LDH) above upper limit of normal (HR, 2.3 [95% CI, 1.3 to 4.5]; *P* = .007), and serum albumin <3.5 g/dL (HR, 1.5 [95% CI, 0.99 to 2.3]; *P* = .056) were independently prognostic. By assigning a score of 1 point each to albumin <3.5 g/dL (HR, 1.5) and age 66-75 years (HR 1.4) and 2 points for age >75 years (HR, 2.6) or elevated LDH (HR, 2.3), four groups with distinct outcomes were observed on the basis of the composite scores. Five-year OS was 93% for the low-risk (score 0), 82% for low-intermediate risk (score 1), 69% for intermediate-risk (score 2), and 55% for the high-risk (score ≥3; *P* < .0001) groups. In the validation cohort (N = 335), the model maintained its prognostic value, with a 5-year OS of 93%, 90%, 75%, and 57% for the four groups, respectively (*P* < .0001).

**CONCLUSION:**

Modified Staging System for WM (MSS-WM), utilizing age, albumin, and LDH is a simple, clinically useful, and externally validated prognostic model that reliably risk-stratifies patients with symptomatic WM into four groups with distinct prognosis.

## INTRODUCTION

Waldenström macroglobulinemia (WM) is an infrequently encountered immunoglobulin M (IgM)–secreting lymphoplasmacytic lymphoma, with a heterogeneous course, ranging from markedly indolent to rapidly progressive disease.^1-3^ The clinical manifestations of active WM vary widely, with symptoms or end-organ damage related to cytopenias, hyperviscosity, amyloid light/heavy amyloidosis (ALH), cryoglobulinemia, histologic transformation (HT), and peripheral neuropathy, among other complications.^4-6^ Our improved understanding of the genomic underpinnings of WM has facilitated identification of molecularly defined subsets and expanded the therapeutic landscape.^7-11^ Robust prognostic models that accurately predict outcomes and potentially aid in rationally developing risk-adapted treatment strategies are needed.

**CONTEXT**

**Key Objective**
Is there a role for a new risk stratification system in patients with active/symptomatic Waldenström macroglobulinemia (WM)?
**Knowledge Generated**
Age (66-75 years and >75 years), serum albumin, and serum lactate dehydrogenase at diagnosis can reliably risk-stratify patients with active WM into four risk groups with distinct outcomes.
**Relevance *(S. Lentzsch)***
The risk stratification model Modified Staging System (MSS)-WM is a simple and robust prognostic tool successfully validated in US and European patients, allowing prognostication without genotyping. In contrast to the currently used revised International Prognostic Scoring System, MSS-WM better differentiates the high and intermediate-risk cohorts from the low/low-intermediate risk groups and can be easily used in clinical practice.*
*Relevance section written by *JCO* Associate Editor Suzanne Lentzsch, MD, PhD.

The International Prognostic Scoring System for WM (IPSS-WM) emerged from a collaborative effort to stage patients requiring treatment into three groups on the basis of five parameters: age, platelet count, hemoglobin, beta-2 microglobulin (β2M), and IgM level.^12^ The original IPSS-WM modeling included patients diagnosed and treated prior to 2002, before the frequent frontline use of chemoimmunotherapy, proteasome inhibitor–based or Bruton tyrosine kinase inhibitor (BTKi)–based regimens, and demonstrated 5-year overall survival (OS) of 87%, 68%, and 36% for the low-, intermediate-, and high-risk groups, respectively.^12^ The IPSS-WM did not assess the issue of non–WM-related deaths.^13,14^ The recently proposed, revised (r) IPSS-WM, on the basis of age, lactate dehydrogenase (LDH), albumin, and β2M, addressed some of the deficiencies but did not examine the impact of molecular parameters, including the myeloid differentiation primary response 88 (*MYD88*)^*L265P*^ mutation, which is now routinely assessed at diagnosis. Moreover, rIPSS-WM could be only partially replicated in the validation cohort and remains to be broadly adopted.^15^ Through this study, we examined the performance of rIPSS-WM in treatment-naïve patients with active WM and propose a refined prognostic model, with external validation in a multi-institutional cohort of patients from the United States and Europe.

## METHODS

Following the Institutional Review Board approval, we included patients with active WM, diagnosed between January 01, 1996, and December 31, 2017, and evaluated consecutively at the Mayo Clinic, Rochester (MCR), Minnesota. This study was conducted in accordance with the Declaration of Helsinki. Each center obtained individual informed consent from patients per institutional requirements. Patients with at least 10% bone marrow lymphoplasmacytic infiltration and a circulating monoclonal IgM protein, causing symptoms and/or laboratory abnormalities requiring initiation of therapy per the Consensus Criteria, were considered to have active WM.^16^ We applied the rIPSS-WM model in patients with data available for all parameters required to assess its utility. We then examined the baseline dichotomized clinical and laboratory parameters affecting OS, with all-cause mortality as the event of interest in the MCR cohort, using a univariate Cox proportional hazard analysis (UVA). To maximize the use of the available data in the setting of varying degree of missing data across variables, the UVA included all patients with available data for the variable under evaluation. This was followed by a multivariate Cox proportional hazard regression analysis (MVA) involving patients with complete data for the variables to identify the independent prognosticators of OS. The variables deemed independently prognostic (*P* ≤ .1) on the MVA were assigned a score proportional to their hazard ratio (HR), wherein those with comparable HR were assigned equal point(s). Little's test was used to assess whether missingness of the data was completely at random and independent of both the observed and the unobserved data, with the null hypothesis that the data were missing completely at random.^17^

The cutoffs for the covariates were selected on the basis of previously established thresholds.^12^ For LDH, the upper limit of normal (ULN) was used as cutoff to ensure generalizability, given the different cutoffs used in the multi-institutional validation cohort. We used a cohort comprising treatment-naïve patients with active WM from five institutions (Data Supplement, Table S1 [online only]) to validate our model. The Kaplan-Meier method was utilized for time-to-event analyses and survival was compared using the log-rank test.^18^ A competing risk analysis was used to validate the model on the basis of the cause of death (cmprsk package, version 2.2-1.1,R).^19^ Deaths occurring from WM progression, HT, ALH, or WM-directed treatment-associated complications, including therapy-related myeloid neoplasm, were considered WM-related and deaths from other causes as competing events; patients alive at last follow-up were censored. The discriminatory power of different models and their relative goodness of fit for predictive score was assessed by computing Harrell's concordance index (dynpred package version 0.1.2,R).

## RESULTS

We identified a cohort of 889 patients with active WM at MCR who had a median follow-up of 8.2 (95% CI, 7.5 to 9) years. Because we could not validate rIPSS-WM in our data set (reported later), we used the MCR cohort data to generate a new prognostic model. Table 1 shows the baseline characteristics of the derivation cohort. Antecedent monoclonal gammopathy of undetermined significance and smoldering WM were documented in 137 (16%) and 168 (19%) patients, respectively. The clinical course was complicated by ALH in 69 (8%) patients, HT in 38 (4%) patients, hyperviscosity in 118 (13%) patients, myelodysplastic syndrome in 17 (2%) patients, and Bing-Neel syndrome in 12 (1.4%) patients. The estimated median OS for the entire cohort is 10.6 (95% CI, 9.7 to 11.4) years. The baseline parameters affecting OS are shown in Table 2. On UVA, age, LDH > ULN, β2M > 3 μg/mL, and albumin <3.5 g/dL at diagnosis of active WM emerged as significant prognosticators for OS. The *MYD88*^*L265P*^ mutation status, known in a subset of patients (n = 296) did not affect OS (median OS, 10 years 95% CI, 8.1 to not reached [NR] among patients with *MYD88*^*WT*^ genotype [n = 65] *v* 11.4 years [95% CI, 10.1 to NR] with *MYD88*^*L265P*^ [n = 231]; HR, 1.3 [95% CI, 0.8 to 2.1], *P* = .33, Data Supplement, Fig S1). The MVA (n = 341) demonstrated serum LDH > ULN, serum albumin <3.5 g/dL, and age groups, 65-75 years and >75 years, at diagnosis as independently prognostic (Table 2).

**TABLE 1.** Baseline Characteristics of the Derivation Cohort With Active/Symptomatic Waldenström Macroglobulinemia

Characteristic	Value
Age at active disease, years, median (range)	66 (31-94)
Age distribution of patients at active disease, years, No. (%)	
≤40	11 (1)
41-50	71 (8)
51-60	206 (22)
61-70	321 (36)
71-80	221 (27)
>80	59 (6)
Sex, male, No. (%)	570 (64)
Race, No. (%)	
Caucasian	837 (97.5)
Non-Caucasian	21 (2.5)
Family history of malignancy in first-degree relatives, No. (%)	355 (40)
Hematologic malignances^a^	81 (9)
WM/IgM MGUS	20 (2.2)
Other lymphomas	24 (3)
Chronic leukemias	17 (2)
Multiple myeloma	12 (1)
Acute leukemias	10 (1)
Solid tumors	274 (31)
Clinical features at active WM, No. (%)	
Constitutional symptoms	506 (57)
Fever	18 (2)
Night sweats	62 (7)
Weight loss	142 (16)
Fatigue	453 (51)
Lymphadenopathy	186 (21)
Splenomegaly	88 (10)
Skin rash	26 (3)
Arthralgia	27 (3)
Laboratory features at active WM, No., median (IQR)	
Hemoglobin (g/dL, n = 647)	10 (9-11.8)
Platelet count (10^9^/L, n = 529)	213 (134-303)
IgM (mg/dL, n = 674)	3,763 (1,890-5,660)
Beta-2 microglobulin (µg/mL, n = 383)	3.4 (2.6-4.9)
Marrow lymphoplasmacytosis (%, n = 637)	50 (30-70)
Free light chain ratio, involved *v* uninvolved, (n = 274)	9 (3-42)
LDH (U/L, n = 385)	144 (116-187)
*MYD88*^*L265P*^ mutation (%, n = 296)	78
Serum albumin (g/dL, n = 480)	3.4 (3.2-3.7)

Abbreviations: IgM, immunoglobulin M; LDH, lactate dehydrogenase; MGUS, monoclonal gammopathy of undetermined significance; MYD88, myeloid differentiation primary response 88; WM, Waldenström macroglobulinemia.

^a^ Two patients had two additional hematologic malignancies apart from WM.

**TABLE 2.** Univariable Analysis of Factors Potentially Affecting Survival of Patients With Active WM

Parameter	Data Available (No.)	Univariate Analysis		Multivariate Analysis (n = 341)	
		Hazard Ratio (95% CI)	*P*	Hazard Ratio (95% CI)	*P*
Age	889				
66-75 years *v* ≤65 years		1.8 (1.5 to 2.3)	**<.0001**	1.4 (0.83 to 2.4)	**.1**
>75 years *v* ≤65 years		3.6 (2.7 to 4.7)		2.6 (1.5 to 4.5)	**.0007**
β2M >3 μg/dL	383	2 (1.4 to 3)	**.0003**	1.1 (0.7 to 1.7)	.8
LDH >ULN	385	1.8 (1.2 to 2.8)	**.007**	2.3 (1.2 to 4.4)	**.007**
IgM >7,000 mg/dL	674	1 (0.7 to 1.4)	.8		
Albumin <3.5 g/dL	480	1.6 (1.2 to 2.1)	**.002**	1.5 (0.99 to 2.3)	**.056**
Lymphoplasmacytosis >50%	638	1 (0.8 to 1.3)	.78		
Hemoglobin ≤11.5 g/dL	647	1.1 (0.9 to 1.5)	.4		
Platelet count ≤100 × 10^9^/L	529	0.9 (0.6 to 1.3)	.52		
FLCR, involved/uninvolved >10	274	1 (0.6 to 1.5)	.89		
MYD88^L265P^	296	0.8 (0.5 to 1.3)	.33		

NOTE. Bold entries depict statistical significance.

Abbreviations: β2M, beta-2 microglobulin; FLCR, serum free light chain ratio; IgM, immunoglobulin M; LDH, lactate dehydrogenase; MYD88, myeloid differentiation primary response 88; ULN, upper limit of normal.

### Prognostic Model Score Calculation

**FIG 1.**
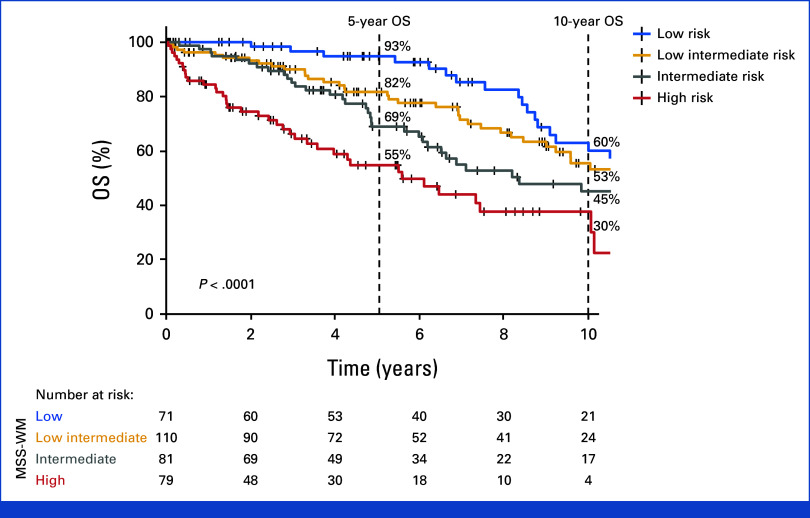
Modified Staging System for Waldenström macroglobulinemia. Simplified prognostic model for OS using age ≤65 years (0 point), 66-75 years (1 point), >75 years (2 points), serum albumin <3.5 g/dL (1 point), and LDH > upper limit of normal (2 points); Low-risk score: 0; low-intermediate risk score: 1, intermediate-risk score: 2, and high-risk score: ≥3. The 5-year OS was 93%, 82%, 69%, and 55% for low-, low-intermediate, intermediate-, and high-risk cohorts, respectively. LDH, lactate dehydrogenase; MSS, Modified Staging System for WM; OS, overall survival; WM, Waldenström macroglobulinemia.

Owing to comparable HR (Table 2), we assigned a score of 1 point each to serum albumin <3.5 g/dL and age 66-75 years and similarly 2 points each to age >75 years and elevated serum LDH, in accordance with their impact on OS. Using this scoring system, a prognostic model was generated with the composite scores ranging from 0 to 5. Patients with the composite scores of 0, 1, and 2 were assigned to the low-risk (n = 71, 21%), low-intermediate risk (n = 110, 32%), and intermediate-risk (n = 81, 24%) groups, respectively. Owing to similar outcomes for patients with the composite scores of 3 to 5 and small sizes of the subcohorts with the composite scores of 4 (n = 14) and 5 (n = 5), these subcohorts were integrated to generate the high-risk group (n = 79, 23%). The estimated median OS for this derivation cohort with complete data (n = 341) was 9.9 (95% CI, 8.5 to 11.4) years. The estimated median OS was 14.6 (95% CI, 9.1 to NR) years, 11.2 (95% CI, 9.1 to 15.2) years, 8.3 (95% CI, 6.4 to 12.2) years, and 5.5 (95% CI, 3.9 to 11) years, for the low-risk, low-intermediate risk, intermediate-risk, and the high-risk groups, respectively; *P* < .0001. The respective 5-year OS rates were 93%, 82%, 69%, and 55%, and 10-year OS rates were 60%, 53%, 45%, and 30% for the low-risk, low-intermediate risk, intermediate-risk, and the high-risk groups, respectively (Fig 1). The proportion of patients with HT and ALH, respectively, was comparable across the four groups (Data Supplement, Table S2).

To assess for any possible selection bias arising from the exclusion of the patients with unavailable data in the MCR cohort, we compared the baseline characteristics and outcome of patients with and without the missing data. Barring a higher proportion of patients with age >65 years in the cohort with the incomplete data (Data Supplement, Table S3), the characteristics were comparable, as was the OS of the two cohorts (Data Supplement, Fig S2). Additionally, data missing at the level of each variable did not affect survival (Data Supplement, Fig S3). The proportion of patients with missing data across the different time periods was similar and is demonstrated in the Data Supplement (Table S4). In missing data analysis, the inability to reject the null hypothesis (*P* = .268) provided sufficient evidence to indicate that the data were missing completely at random.

### Prognostic Model Validation

**FIG 2.**
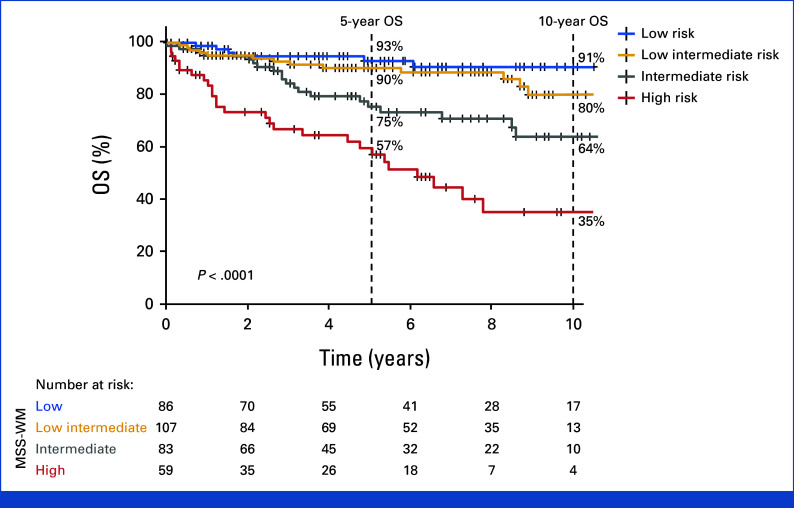
Validation of the MSS-WM prognostic model in an external cohort reveals a 5-year overall survival of 93%, 90%, 75%, and 57% for the low-risk, low-intermediate risk, intermediate-risk, and high-risk cohorts, respectively. MSS, Modified Staging System for WM; WM, Waldenström macroglobulinemia.

We next validated the proposed model, henceforth called the Modified Staging System for WM (MSS-WM), in an external (validation) cohort (N = 335), with a median follow-up of 6.3 (95% CI, 5.5 to 7.2) years. Eighty-six (26%) patients were stratified as low-risk, 107 (32%) patients as low-intermediate risk, 83 (25%) patients as intermediate-risk, and 59 (18%) patients as high-risk. The 5-year OS rates were 93%, 90%, 75%, and 57% and 10-year OS rates were 91%, 80%, 64%, and 35% for the low-risk, low-intermediate risk, intermediate-risk, and high-risk cohorts, respectively (*P* < .0001, Fig 2). Similar to the derivation cohort, the *MYD88* genotype did not significantly affect the OS of the validation cohort (5-year OS of 64% *v* 81% for the *MYD88*^L265P^ and *MYD88*^WT^ genotype, respectively, *P* = .10).

### Performance of Existing Prognostic Models for WM

**FIG 3.**
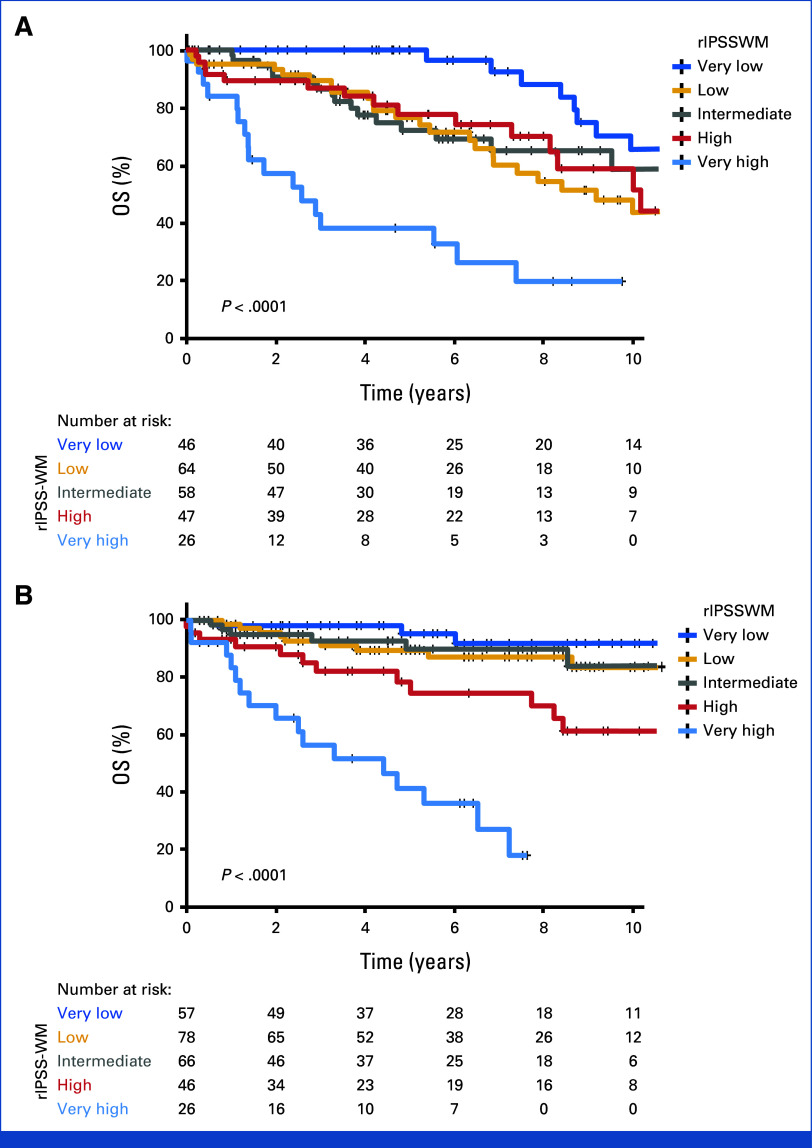
Failure to validate rIPSS-WM in two separate cohorts: (A) The rIPSS model when applied to the MCR cohort demonstrates 5-year OS of 96%, 76%, 72%, 77%, and 32% for the very low-, low-, intermediate-, high-, and very high-risk cohorts of rIPSS-WM, respectively, with intertwined survival curves for the low-, intermediate-, and the high-risk groups. (B) Application of the rIPSS-WM model to the external cohort demonstrates 5-year OS of 95%, 89%, 90%, 75%, and 36% for the very low-, low-, intermediate-, high-, and very high-risk cohorts of rIPSS-WM, with poor discrimination among the lower-risk cohorts. MCR, Mayo Clinic, Rochester; OS, overall survival; rIPSS, revised International Prognostic Staging System for WM; WM, Waldenström macroglobulinem.

**FIG 4.**
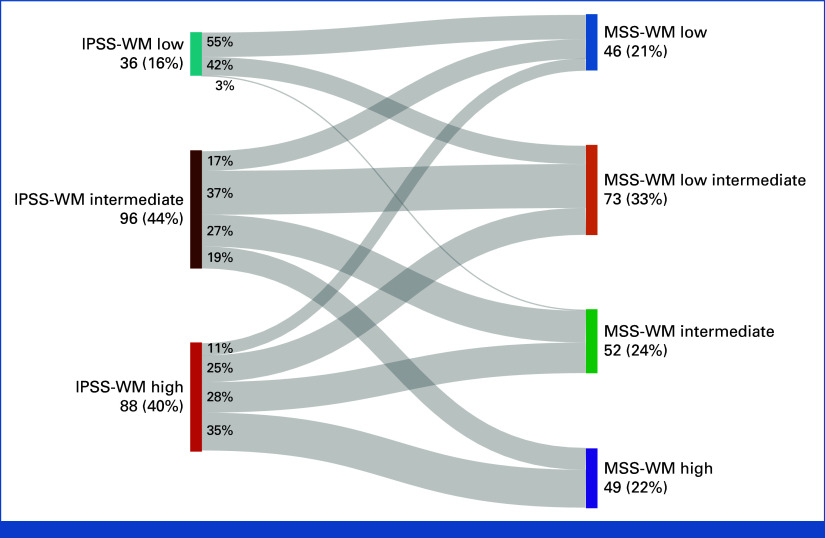
Sankey diagram showing a high-level view of the redistribution of the patients from the IPSS-WM risk categories to the MSS-WM risk groups: Among 220 patients, a substantial proportion from the high-risk IPSS-WM (65%, 57/88) were downstaged as low-, low-intermediate, or intermediate-risk by MSS-WM, whereas 44% (16/36) of patients in low-risk IPSS-WM were upstaged as low-intermediate or intermediate-risk by MSS-WM. Similarly, a sizable proportion of patients from the IPSS-WM intermediate group were reclassified to the MSS low (17%, 16/96) and MSS high (19%, 18/96)-risk groups. Int, intermediate risk; IPSS, International Prognostic Staging System for WM; Low-Int, low-intermediate risk; MSS, Modified Staging System for WM; WM, Waldenström macroglobulinemia.

The rIPSS-WM model was examined in our MCR (derivation) and validation cohorts.^15^ Patients with available data (n = 241) in the derivation cohort were risk stratified into very low- (n = 46), low- (n = 64), intermediate- (n = 58), high- (n = 46), and very high-risk groups (n = 27), with the respective 5-year OS rates of 96%, 76%, 72%, 77%, and 32%. Although the concordance index of 0.67 (95% CI, 0.61 to 0.73) for the rIPSS-WM model was similar to that of the derivation cohort of MSS-WM model (concordance index 0.68, 95% CI, 0.63 to 0.73), the survival curves of the patients in the low-, intermediate-, and high-risk groups were overlapping (Fig 3A). Similarly, in the validation cohort, patients with available data for rIPSS-WM (n = 273) demonstrated poor discrimination in the very low-, low-, and intermediate-risk groups (Fig 3B). Next, we assessed the performance of the IPSS-WM in the MCR cohort of patients with data available for IPSS-WM–based stratification (n = 319). The 5-year OS was 92% (95% CI, 84 to 99) for the low-risk group, 76% (95% CI, 68 to 85) for the intermediate-risk group, and 69% (95% CI, 60 to 77) for the high-risk IPSS-WM group (*P* = .009; concordance index 0.58 [95% CI, 0.52 to 0.63], Data Supplement, Fig S4). Among patients with data available to calculate MSS-WM and IPSS-WM (n = 220), a substantial proportion of the high-risk IPSS-WM (65%, 57/88) were down-staged as low, low-intermediate, or intermediate risk by MSS-WM, whereas 44% (16/36) of patients in low-risk IPSS-WM were upstaged as low-intermediate or intermediate risk by MSS-WM. Similarly, a sizable proportion of patients from the IPSS-WM intermediate group were reclassified in the MSS-WM model (Fig 4). The Data Supplement (Fig S5 and Table S5) shows the redistribution of patients and the distinct outcomes of the patients who were reclassified by the MSS-WM within each stratum of the IPSS-WM (Fig S6). By contrast, the IPSS-WM did not identify any subcohorts with dissimilar outcomes within each MSS-WM cohort (data not shown).

### Cause of Death in WM

Among 360 (40%) patient deaths in the derivation cohort, the cause was attributable to WM in 230 (63.9%), with unrelated second primary malignancies (n = 29; 8%), cardiovascular (n = 16; 5%) and neurologic issues (n = 16; 5%) being the most frequent causes for non-WM related deaths. The cause was unclear in 43 (12%) patients but likely unrelated as WM was in remission around the time of death. Using competing risk analysis, the proposed staging model demonstrated a 5-year OS of 95% for the low-risk cohort, 85% for the low-intermediate risk cohort, 85% for the intermediate-risk cohort, and 71% for the high-risk cohort (*P* = .0001). The proportional distribution of deaths from WM by age is demonstrated in the Data Supplement (Table S6).

### Treatment Patterns

Single-agent rituximab was the commonest frontline therapy (Data Supplement, Table S7) used (n = 277, 33%) in the derivation cohort, followed by rituximab-alkylator combinations (n = 234; 28%) and alkylator monotherapy (n = 167; 20%). A steady increase in the rituximab-alkylator combination as the frontline therapy was observed over the study period: 16% in 2001-2005, 29% in 2006-2010, 44% in 2011-2015, and 55% beyond 2015 (Data Supplement, Fig S7). Patients who received rituximab during their disease course (n = 737) had a superior OS compared with rituximab nonexposed patients (n = 98; 11.2 *v* 4.7 years; *P* < .0001). The MSS-WM held its discriminative ability in patients exposed to rituximab-based therapies during the disease course (*P* < .0001, Data Supplement, Fig S8) in addition to the non–BTKi-treated cohort (*P* < .0001, Data Supplement, Fig S9). However, within the study time frame, only a small fraction of the evaluable patients (n = 57, 17%) were exposed to BTKi, precluding the assessment of MSS-WM in this subset.

## DISCUSSION

On the basis of our findings, we propose a simpler, externally validated staging system, the MSS-WM,^40^ which risk- stratifies patients into four distinct groups, demonstrating 5-year OS rates ranging from 55% to 93% and clearly delineating a subset with an outstanding outcome, despite the lack of curative therapies.

Low serum albumin is a marker of patients' overall health and poor nutritional status, reflecting reduced hepatic synthesis, mediated in part by high levels of circulating interleukin-6 and other cytokines in WM, and is independently prognostic.^20,21^ Additionally, low albumin may occur in ALH, a complication of WM with distinctly poorer outcomes.^22^ Not surprisingly, elevated LDH, a marker of high cell turnover and aggressive disease biology, which has been incorporated in other staging systems of related lymphoproliferative malignancies, emerged as independently prognostic.^13,23^ Similar to the previous studies, age remained a crucial determinant of prognosis. Strikingly, even among patients with age >75 years, most deaths were deemed WM-related. These findings are consistent with the previous data showing a reduced survival rate for elderly patients (older than 75 years at the time of diagnosis of WM) compared with the age- and sex-matched US population.^24^ Previous studies with limited strength and reproducibility have identified various prognostic factors, including hemoglobin, β2M, and IgM concentration, among others, paving the way for the IPSS-WM, the most consistently used prognostic tool since its development.^23,25,26^ Many of these variables were, however, not prognostic in our cohort.

The MSS-WM model stems from the dichotomization of continuous variables, using the previously established cutoffs in the IPSS-WM model to maintain uniformity for optimal comparison. The IPSS-WM stratifies the bulk of patients (approximately 75%) as intermediate or high risk. Whereas MSS-WM, despite using fewer variables, captures a greater degree of patient heterogeneity as reflected by increased 4-tiered segregation of patients at similar proportions within the respective derivation and the validation sets: low risk (21%, 26%), low-intermediate risk (32%, 32%), intermediate risk (24%, 25%), and high risk (23%, 18%). The IPSS-WM–based prediction in our MCR cohort demonstrated a 5-year OS rate range of 69-92%, with the high-risk group exhibiting an appreciable improvement in the outcome compared with the historical data (5-year OS of 36%). The concordance index, a metric to evaluate the predictive models' capacity to discriminate, was higher for MSS- WM (0.68) than the IPSS-WM model (0.58) in the same cohort. Importantly, the MSS-WM could capture the heterogeneity of outcomes within each stratum of the IPSS-WM.

Notably, the rIPSS-WM model poorly discriminated the intermediate-risk groups, with overlapping survival curves both in our derivation and validation cohorts, a finding previously observed in the smaller validation cohort (n = 228) of the original rIPSS-WM study.^15^ However, the comparable concordance indices for the rIPSS-WM and MSS-WM likely reflect the wide separation of the lowest- and highest-risk groups, observed in the former model. Unlike the rIPSS-WM model, MSS-WM reliably differentiates the high- and intermediate-risk cohorts from the low/low-intermediate risk groups in the validation cohort. The survival curves of the low-risk and the low-intermediate risk groups of the MSS-WM validation set will probably diverge further as the follow-up duration approximates that of the training set, resulting in more events in the low-intermediate group.

One of the limitations of the IPSS-WM model was the lack of data regarding the cause of death and its impact. In the rIPSS-WM modeling, 22% deaths were WM-unrelated.^13^ In our cohort, 37% of the deaths were deemed WM-unrelated. Therefore, given the advanced age at presentation, we performed a competing risk analysis to accurately assess OS and the MSS-WM model held its discriminative ability.

The rates of ALH and HT, complications typically associated with inferior survival in patients with WM,^22,27^ were similar across the four risk groups (Data Supplement, Table S2) and comparable with the previously reported data, making them unlikely to skew outcomes.^28,29^ Mirroring previous studies, nearly one in five patients with active WM in our cohort had antecedent smoldering WM.^30,31^ To avoid the potential impact of the lead-time bias arising from an incidental diagnosis, we estimated OS from the development of active/symptomatic WM rather than the diagnosis of smoldering WM.

Mutated *MYD88* has shown a contrasting impact on OS in previous studies.^32-34^ However, this molecular information was not examined for the creation of the rIPSS-WM model. In our large derivation and validation cohorts, the locally assessed *MYD88*^*L265P*^ genotype did not significantly influence outcomes. Delineating the impact of a specific therapy/regimen on survival is challenging as, eventually, a sizable proportion of survivors receive most of the frequently used therapies during the relapsing-remitting course of WM. Although exposure to rituximab as monotherapy or combination improved survival, this gain may be partly attributable to the non–rituximab-exposed cohort belonging to an earlier era, with inferior supportive care and limited access to novel effective treatments. Nonetheless, our rituximab nonexposed cohort, accounting for a small subset, did not affect the generalizability of MSS-WM.

Recognizing the inherent limitations of a retrospective analysis, we attempted to overcome the biases introduced because of the unavailable data by comparing outcomes and the baseline characteristics of the cohorts with and without the missing data. Our findings were largely similar in the two cohorts. Data regarding the CXC motif chemokine receptor 4 (*CXCR4*) mutation, associated with resistance to certain treatments, were absent in most patients, precluding its incorporation into the model. Regardless, in contrast to the *MYD88*^*L265P*^ assessment, incorporation of *CXCR4* mutation(s) status would be challenging because of the costs and complexity of the assay, involving more than 40 different mutations, and consequently sparse testing outside of academic centers.^35,36^ In the future, a targeted-approach, using a polymerase chain reaction–based assay to examine the commonest mutation, *CXCR4*^*S338X*^, may render the test results more readily available to augment the proposed model, if found to be independently prognostic. Owing to the shorter follow-up since the approval of the BTKi for WM, a small fraction of patients in our cohort have received this class of agents as primary therapy. Therefore, the applicability of MSS-WM in the BTKi era would require reexamination in the future, in a large cohort with a protracted follow-up. Additionally, MSS-WM merits prospective validation in clinical trials and evaluation as a prognostic tool to guide clinical decision making.

Notwithstanding these limitations, MSS-WM represents a robust multi-institutional effort, involving a large cohort, with a respectable follow-up. Incorporation of the routinely obtained variables enhances its applicability in clinical practice. MSS-WM provides a platform for future refinement as more genetic and molecular data pertaining to deletion (del)6q, del17p, and mutations in *TERT*, *CXCR4*, and *TP53* are gathered in trials.^37-39^

In summary, MSS-WM is a simple, externally validated, robust, risk-stratification model based on patients' age, serum albumin, and serum LDH, which reliably captures the prognosis of previously untreated patients with active WM.
